# Microstructural Evolution and Damage Mechanism of Water-Immersed Coal Based on Physicochemical Effects of Inorganic Minerals

**DOI:** 10.3390/ma17225579

**Published:** 2024-11-15

**Authors:** Xuexi Chen, Zijian Liu, Tao Li, Jingyi Ma, Jiaying Hu

**Affiliations:** School of Mine Safety, North China Institute of Science and Technology, Langfang 065201, China; xuexichen1210@163.com (X.C.); zijianliu010@163.com (Z.L.);

**Keywords:** water-immersed coal, inorganic mineral, microstructure, mechanical property, pore evolution

## Abstract

Coal seam water injection technology enables seam permeability enhancement and facilitates outburst risk reduction. This study investigated the microscale effects of water infiltration on coal and the evolution mechanisms of its mechanical properties. To this end, we systematically analyzed dynamic changes (such as mineral composition, pore structure, and mechanical performance) in coal soaked for various durations using X-ray diffraction, low-field nuclear magnetic resonance (NMR), and uniaxial compression testing. The results indicate: (1) the coal NMR T_2_ spectrum displays three characteristic peaks, corresponding to rapid water absorption, uniform transition, and stabilization stages of soaking traditionally divided according to peak area variation trends. (2) The coal strength decreases with progressive soaking, influenced by water content, pore volume, mineral composition, etc. Its compressive strength and elastic modulus drop by 22.4% and 19.5%, respectively, compared to the initial values. (3) The expansion of clay minerals during immersion reduces average pore size. In contrast, quartz particle displacement, pore water movement, and soluble mineral dissolution increase pore volume, reducing the overall structure strength. (4) The dominant factors driving the degradation of mechanical properties vary across immersion stages, including water content and specific mineral concentration. This work offers new insights into how hydraulic technology alters coal seams, providing theoretical support for optimizing water injection strategies in the seam.

## 1. Introduction

Coal resources still represent an impressive share of the global energy mix [1]. In countries such as Poland, China, India, and the United States, coal is a primary source of energy consumption and a pillar of energy security due to its abundant reserves and economic viability. The ongoing war in Ukraine and rising energy prices have raised growing energy security concerns across Europe and beyond, heightening the urgency for a transition to renewable energy [2]. Despite the rapid development in this sector, achieving the goal of coal replacement in the short term remains challenging. In this context, studying the coal resources’ physical and chemical properties under complex conditions still holds importance. Such investigations contribute to enhancing mining efficiency, guiding the efforts to reduce dependence on coal, and advancing energy structure transformation. The increasing depletion of shallow coal resources, compounded by the rising demand and advancements in engineering technology, has led to a shift towards deep mining. Increased coal extraction depths and the complexity of geological conditions present escalating challenges to coal mine safety [3,4,5,6,7,8]. In particular, the inherent properties of low-permeability outburst-prone coal seams without protective layers further increase the difficulty and risks of mining practices [9]. Against this backdrop, coal seam water injection technology has been widely adopted to enhance coal seam permeability and reduce outburst risks [10,11]. Water, a key factor influencing the coal properties, has been a focal point of research.

Researchers have conducted extensive experimental studies to address the issue of coal weakening due to water immersion. Zhuang [12] investigated the impact of water on the coal samples’ seepage characteristics throughout the stress–strain process and observed a noticeable decrease in the compressive strength after water immersion. Lu [13] studied the crack propagation behavior of coal under uniaxial compression at varying moisture content levels. Their results showed that increasing moisture content reduced coal brittleness and enhanced plasticity. Furthermore, the results from Han’s [14] long-term water immersion experiments reveal the damage progression; moreover, the established constitutive model of water-soaked coal samples demonstrated that structural damage to coal intensified with prolonged immersion duration. These studies typically use X-ray diffraction (XRD) and scanning electron microscopy to analyze the mineral composition and microscopic pore structure of coal. Zhang [15] analyzed the effects of long-term water immersion on the two properties. The findings showed that the overall porosity of coal samples increased over time; in contrast, the mineral composition decreased in volume fraction, mainly due to the dissolution of some clay minerals in water. Zhang [16] further pointed out water played a crucial role in expanding coal pores and forming macropores based on microstructural analysis. Qin [17] demonstrated that changes in unfrozen water content within coal during freeze-thaw cycles in liquid nitrogen affected pore structure and increased pore volume. DU [18] studied the effects of water on the coal pore structure under different temperature and pressure conditions. The results showed that the micropore and mesopore structures underwent noticeable changes, and the fractal dimension exhibits a weak positive correlation with carbon content. Nuclear magnetic resonance (NMR) technology has been a popular tool for characterizing coal pore structures. This technique can obtain information on pore structure changes and fluid migration within the coal structure by measuring the relaxation characteristics of protons in fluids (such as water). Zhai [19] analyzed the porosity, pore size distribution, and connectivity of coal, revealing the pore structure characteristics of different coal samples. Guo [20] incorporated X-CT scanning technology into a multi-scale quantitative study of coal pore structure, providing novel insights into coal permeability and microstructural changes. The dynamic changes during the coal-water immersion process have also attracted much scholarly attention. For instance, through NMR analysis of the water absorption, Feng [21] found that the T_2_ spectrum of coal samples expanded rapidly during the initial stages of water absorption, forming more microcracks in the later stages. Additionally, Reyila [22] reported that the number of micropores in coal largely increased after water saturation treatment, thereby enhancing the porosity and permeability. The above studies mainly focus on (1) the weakening of coal strength, (2) the characterization of coal pore structure at the microscopic scale using NMR, and (3) the damage mechanism of coal under brief water immersion experiments. Despite existing insights, the intrinsic relationship between the microstructural evolution of coal containing inorganic minerals and its weakening of strength under long-term water immersion remains a research gap.

Due to variations in coal formation environments and coalification degrees, the moisture content, mineral composition, and porosity of coal, as well as the impact of soaking durations on these properties, vary greatly. For this reason, this study focused on bituminous coal and conducted NMR experiments to monitor the entire soaking process. The immersion process was divided into three stages based on the microscopic pore evolution patterns. Additionally, XRD and mechanical tests were incorporated to explore the dynamic alterations in the mineral composition, pore structure, and mechanical properties of coal under varying water immersion durations. The objective is to reveal the micro-physicochemical changes in coal under long-term water immersion and their impact on macroscopic mechanical properties, supporting and guiding the optimization of coal seam water injection technology.

## 2. Materials and Methods

### 2.1. Sample Preparation

The coal samples were collected from a typical low-permeability coal seam at Xiangshan Coal Mine, Hancheng, Shaanxi Province, China. All the coal was cored along bedding planes from the same seam to ensure sample representativeness and data integrity. According to the standards of the International Society for Rock Mechanics, these samples were processed into cylindrical shapes measuring 50 mm in diameter and 100 mm in height. In addition, diameter deviations were controlled to within 0.3 mm, and those for end-face parallelism were within 0.05 mm, guaranteeing perpendicular alignment to the axis. Mechanical disturbances were minimized during the preparation to preserve the original physical properties of coal. Given coal’s inherent fragility, 200 samples were prepared, and 140 high-quality samples with consistent mass and appearance were selected for subsequent experiments.

To minimize the impact of coal sample heterogeneity on the experimental results, their initial characteristics were evaluated prior to the immersion test. For the day 0 samples, the average mass was approximately 250 g, with a moisture content of approximately 22% and a compressive strength of about 10.4 MPa. The primary inorganic minerals and corresponding relative abundance were identified, including kaolinite (48.7%), quartz (21.9%), and pyrite (17.4%).

### 2.2. Water Immersion Test Design

**Figure 1:**
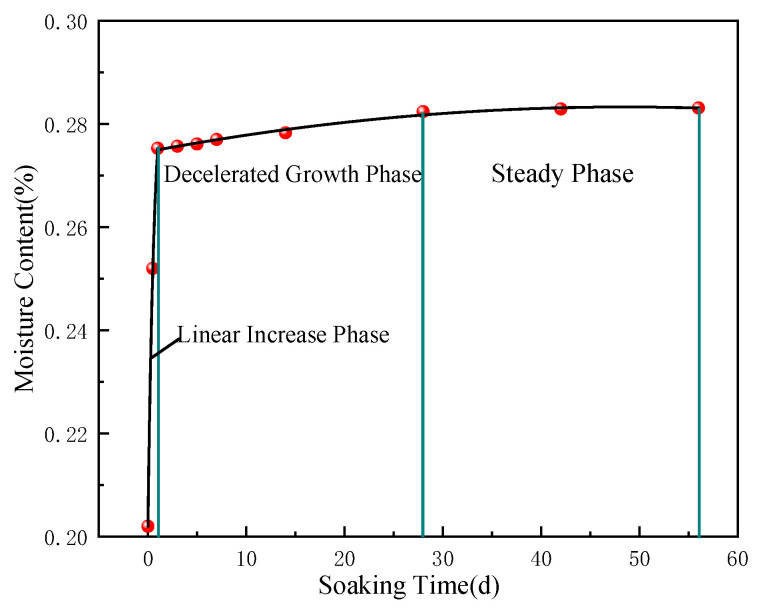
Changes in moisture content of coal samples under various soaking duration conditions.

To investigate the effects of water immersion on the coal microstructure and mechanical properties, the samples were immersed in mine water at room temperature. The mass of each sample was periodically recorded in this process to calculate moisture content. As shown in Figure 1, moisture content increased linearly over the first day, followed by an exponential rise, and eventually reached saturation at approximately 28 days.

Based on this trend, three critical states were identified: dry (0 days), near-saturation (1 day), and full saturation (28 days). Additional intervals at 3, 7, 14, and 56 days were added to facilitate seamless analysis of immersion effects on the coal structure, resulting in seven immersion durations.

### 2.3. Experimental Testing Procedures

**Figure 2:**
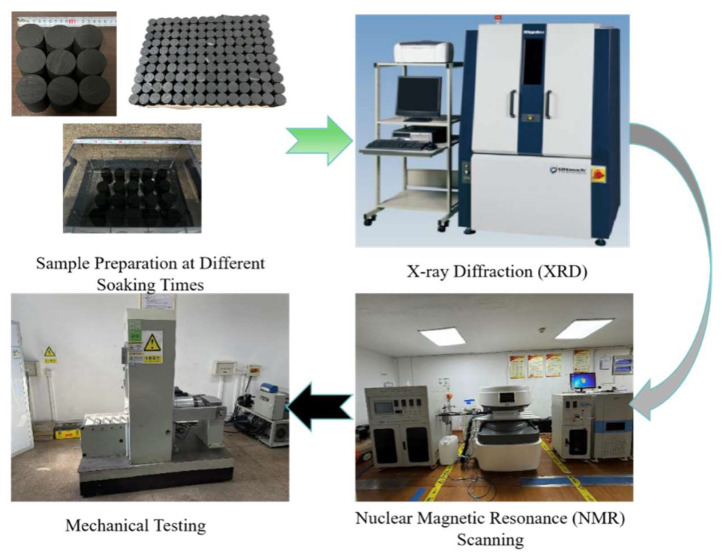
Graphical representation of Experimental Methodology.

The experiments utilized a Bruker D8 ADVANCE (Bruker Corporation, Billerica, MA, USA) multifunctional X-ray diffractometer, a MacroMR12-150H-I NMR imaging system (Niumag Corporation, Suzhou, China), and an RMT-301 rock and concrete mechanical testing machine (Institute of Rock and Soil Mechanics, Chinese Academy of Sciences, Wuhan, China), as presented in Figure 2.

The 140 coal samples were categorized into seven groups, each containing 20 samples, with approximately eight samples per group as backups. Two samples from the last group were selected for XRD and NMR analysis. Excluding the first sample group, the rest were immersed in mine water for 1, 3, 7, 14, 28, and 56 days. The samples were promptly removed after immersion, gently dried with non-woven fabric to remove surface moisture, sealed with plastic wrap, and subjected to subsequent tests without delay.

In the mechanical tests, three replicate experiments were conducted for each soaking duration and confining pressure condition to minimize the impact of sample heterogeneity. Each coal sample was assigned a unique identifier based on its soaking duration and test group to ensure traceability. For instance, the identifier “D-0-1” denotes a sample examined after being soaked for 0 days in a uniaxial compression test, and “S-0-1” represents the counterpart measured after 0 days of soaking in a triaxial compression test. The final digit in the identifier indicates the specific replicate within the corresponding group.

The selected samples at specific intervals (0, 1, 3, 7, 14, 28, 42, and 56 days) underwent mineral composition analysis using the Bruker D8 ADVANCE XRD to quantify major inorganic minerals, such as kaolinite and pyrite, thereby revealing their evolution during immersion. Additionally, NMR analyses were conducted at 0, 1, 7, 14, 28, and 56 days to investigate the effects of water immersion on pore structure, elucidating pore evolution across various scales. Lastly, uniaxial and triaxial compression tests were performed to measure changes in compressive strength and elastic modulus under varying immersion duration conditions, enabling a comprehensive assessment of water immersion’s impact on the coal’s mechanical properties.

## 3. Experimental Results and Analysis

### 3.1. Evolution of Inorganic Minerals

**Figure 3:**
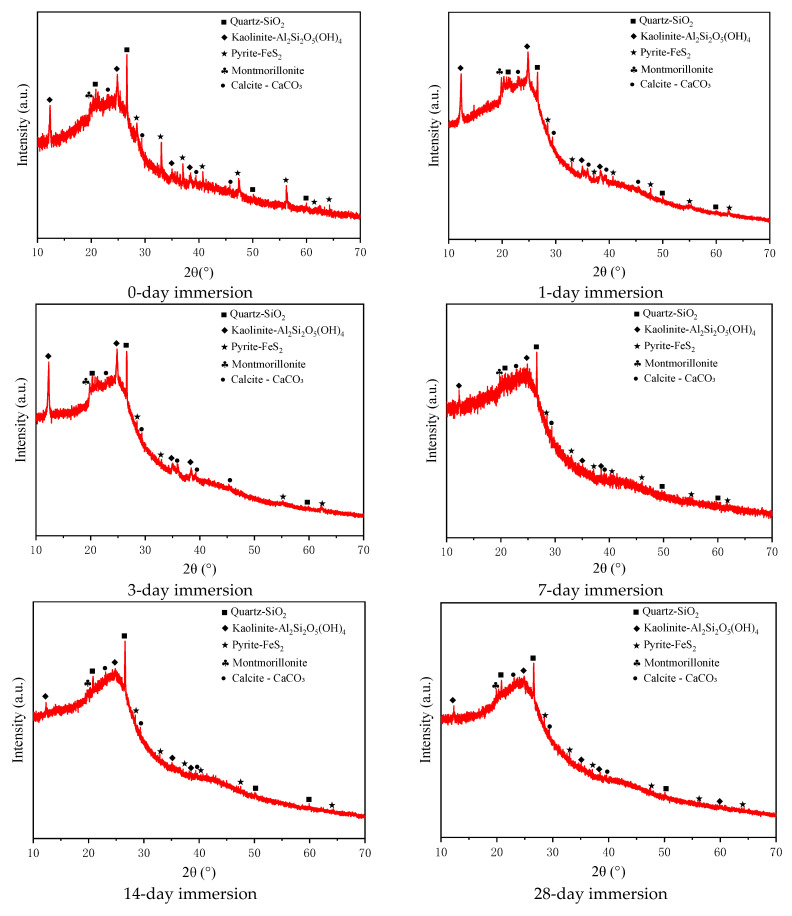

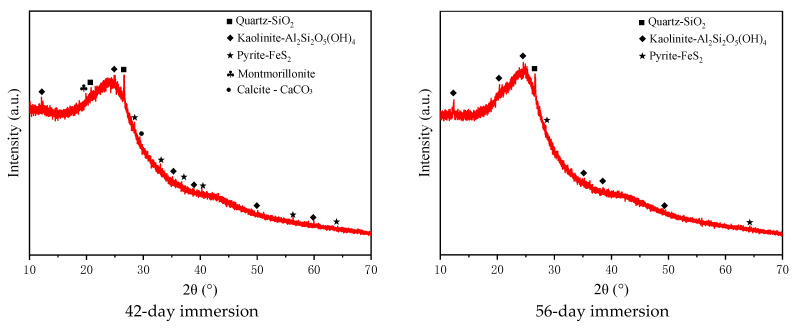
XRD analysis of coal samples at different soaking durations.

The mineral composition of coal samples subjected to water immersion for varying durations was analyzed using XRD (Figure 3). The results indicated that kaolinite, quartz, and pyrite are the primary inorganic minerals in the coal. Additionally, small levels of montmorillonite and calcite were detected. The content of these minerals varies noticeably with progressive immersion.

It is important to determine the relative content of different mineral phases in coal samples and observe the variation trends of these minerals throughout soaking. For this purpose, we quantitatively analyzed the mineral composition, and the results are summarized in Table 1.

Kaolinite is a highly hydrophilic layered silicate mineral. XRD data indicated that its content increased from 48.7% to 60.2% during the early stages of soaking (0–3 days), possibly due to enhanced hydration [23]. In addition, this particulate clay mineral has a hydrophobic siloxane surface and a hydrophilic gibbsite-like surface. Kaolinite crystals aggregate tightly via hydrogen bonding, allowing water to quickly penetrate through adsorption, increasing the mineral’s volume. After seven days, its content decreases to 41.3%. This may be due to the collapse of some interlayer structures induced by prolonged hydration or intensified competition with other minerals for adsorption [24]. At the soaking duration of 56 days, the relative content of kaolinite increases to 77.2% with the dissolution of pyrite and calcite.

The content of the chemically inert mineral (i.e., quartz) rises from 21.9% on day 0 to 45.4% on day 28 and then decreases to 22.6% by day 56. This change is mainly attributed to the dissolution of pyrite and calcite [25], increasing the relative content of quartz. XRD results show that the diffraction peak intensity of quartz remains stable, indicating no marked structural changes [26].

The changes in pyrite and calcite primarily arose from dissolution. Pyrite content rapidly decreased from an initial 17.4% to 0.2% by day 56. This decline suggests oxidative decomposition in the presence of water and oxygen, forming other minerals or amorphous products [27]. In contrast, calcite content experiences a decline by day 42 compared to day 0 (6.1% vs. 1.8%), and it is completely dissolved by day 56. The dissolution of these two minerals largely influenced the coal pore structure.

Montmorillonite exhibits slight swelling during water absorption for 0–3 days, with its content increasing from 5.9% to 7.2%. With progressive immersion, particularly after 14 days, montmorillonite disintegration occurs, leading to its content falling to 1.0% by day 42.

It can be concluded that water immersion markedly impacts the minerals in coal, and the resulting changes influence the pore structure, delivering indirect effects on the coal’s macroscopic mechanical properties. The swelling of kaolinite leads to pore expansion. By comparison, the dissolution of pyrite and calcite accelerates structural degradation. The disintegrated montmorillonite further degrades the mechanical properties of samples. These mineral changes reveal the degradation mechanisms of coal under water immersion conditions.

### 3.2. Distribution of NMR T_2_ Spectra

NMR is an analytical technique based on the physical phenomenon of nuclear spin in a magnetic field. It is frequently used to explore molecular structure, material composition, and internal microstructural features. NMR enables the investigation of changes in pore structure inside coal, primarily due to its sensitivity to the relaxation time of water molecules within pores. By detecting the distribution and dynamic behavior of water in coal samples, NMR captures detailed information on the pore structural characteristics.

#### 3.2.1. Distribution of Transverse Relaxation Time (T_2_)

Fluid molecules in the pores of soil and rocks typically experience three relaxation mechanisms: free, surface, and diffusion processes. Specifically, free relaxation and surface relaxation affect both T_1_ and T_2_ relaxation. In contrast, diffusion relaxation only occurs in the presence of a gradient magnetic field and influences exclusively T_2_ relaxation [28,29]. Accordingly, T_2_ relaxation can be expressed as the sum of the three relaxation behaviors. The total transverse relaxation rate in NMR is obtained using Equation (1).

(1) $$\frac{1}{T_{2}}=\frac{1}{T_{2f}}+\frac{1}{T_{2s}}+\frac{1}{T_{2k}}$$

where *T*_2f_ represents free relaxation, corresponding to the T_2_ relaxation time of pore fluids in sufficiently large containers. *T*_2s_ denotes the T_2_ relaxation time caused by surface relaxation. *T_2k_* indicates the T_2_ relaxation time induced by diffusion under a gradient magnetic field.

*T_2k_* and *T_2f_* can be neglected, given that their values are much smaller than *T*_2s_. Thus, the T_2_ relaxation of rocks can be approximately considered equal to the surface relaxation T_2s_, which can be calculated as follows:

(2) $$\frac{1}{T_{2}}\approx \frac{1}{T_{2s}}=\rho {\left(\frac{S}{V}\right)}_{\mathrm{pore}}$$

where *ρ* represents the surface relaxation strength, and $\rho {\left(\frac{S}{V}\right)}_{\mathrm{pore}}$ refers to the specific surface area of the pores.

According to the relationship between pore radius and transverse relaxation time in Equation (2), a prolonged relaxation period enlarges the pore radius [30]. This relationship allows for quantitatively analyzing the pore size distribution in coal samples based on the T_2_ spectra, with each peak corresponding to a specific porosity. Following the peak partitioning method, from left to right, the first peak in the T_2_ spectrum corresponds to small pores in the coal, the second peak represents the medium pores, and the third peak is related to large pores and fractures [31]. Coal samples typically contain pore structures of various scales, and the T_2_ relaxation time varies depending on the pore size. On this basis, the T_2_ relaxation time in NMR spectra reflects the pore size distribution within the rock. The integral of the T_2_ spectrum signal represents the pore volume, and signal continuity indicates the connectivity of the microscopic pore structure within the rock.

#### 3.2.2. Characteristics of T_2_ Spectra and Pore Structure Changes

**Figure 4:**
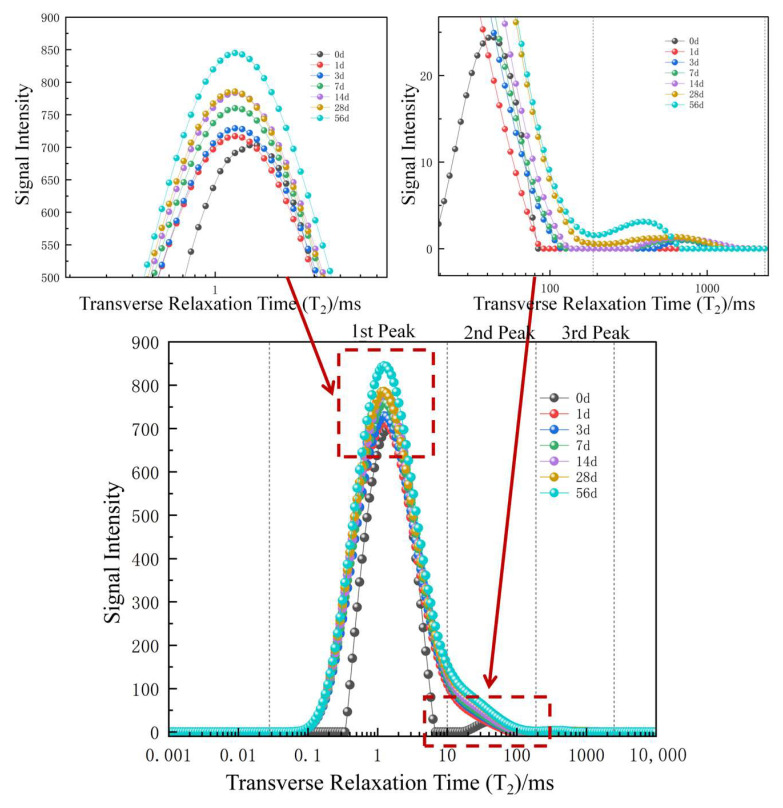
T_2_ Spectra of Coal at Different Soaking Durations.

Figure 4 plots the T_2_ spectra of coal samples at different soaking durations. The T_2_ distribution pattern can be divided into three peaks according to the peak partitioning method. Specifically, peak 1 represents the small pores, peak 2 is associated with the medium pores, and peak 3 indicates large pores and fractures. The relaxation time ranges from 0.1 to 10 ms for small pores, from 10 to 100 ms for medium pores, and exceeds 100 ms for large pores and fractures.

**Figure 5:**
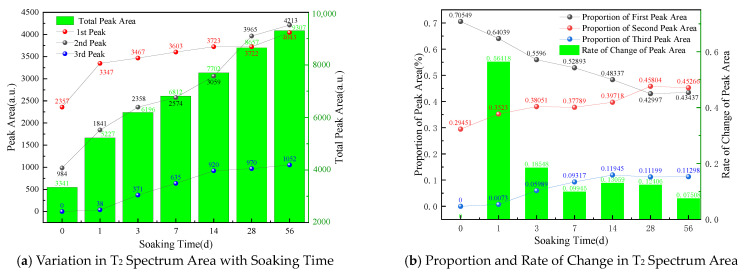
Variations in T_2_ Spectrum Area and Proportion of Coal Samples at Different Soaking Durations.

When soaking time increases, the changes in the T2 spectral peaks reflect a pronounced evolution in the pore structure [32,33,34,35]. Figure 5 shows the variations in the peak area of the coal T_2_ spectra under various soaking duration conditions. In the early stages of soaking, the internal pore volume is relatively small when the coal sample is in a dry state. Specifically, small pores represent 71.1% of the total volume, and the proportion of medium pores is 28.9%. No large pores are observed at this stage.

From Figure 5, it can be seen that the spectral areas of pores continuously increase during immersion from day 0 to 58 despite variations in the proportion. Three stages are identified in this process. The first stage is from 0 to 1 day, characterized by a noticeable growth in the pore volume (up to 56.4%). During this period, the proportion of medium-sized pores rapidly increases, while that of small pores decreases. This difference indicates that considerable micropores are formed, expanded, and connected, contributing to newly generated medium-sized pores. Additionally, the leftward shift of the first peak indicates a reduction in the average pore size of small pores. This may be related to the local collapse of pore walls due to rapid water infiltration or the deposition of mineral particles within pores caused by hydration, which causes originally larger small pores to shrink. Combined with Figure 1, it can be inferred that pore changes are primarily driven by water absorption in the current stage, and the variations in pore and fracture structures are dominated by water content.

The second stage occurs between 1 and 28 days, featuring a slower rate of changes in pore proportions than the first stage. The proportion of small pores experiences an ongoing decrease, from 64% to 43%. In contrast, medium-sized pores gradually outnumber small pores in proportion, which elevates from 35% to 46% and eventually becomes dominant. The proportion of large pores increases from 0.73% on day 1 to 11.99% on day 28, and the third peak shifts leftward, indicating additional decreases in their average pore size. Moreover, the third stage exhibits a continued increase in spectral area, and the growth rate is substantially lower than in the first two stages. The proportions of small, medium, and large pores stabilize at approximately 43%, 46%, and 11%, respectively. During this stage, the three peaks become fully connected, and the distribution of T_2_ spectra tends to become continuous. The differences between pores and fractures of different sizes diminish, and the pore-to-pore connectivity increases, forming a more uniform pore network structure dominated by medium-sized pores.

By combining the above analysis with Figure 1, it can be indicated that the coal samples reach saturation during this phase, and the evolution of its pore structure is dominated more by changes in minerals contained than by water content. Based on the above analysis, the coal-water immersion can be divided into rapid water absorption, uniform transition, and stabilization phases.

### 3.3. Analysis of Mechanical Test Results

#### 3.3.1. Analysis of Uniaxial Compression Test Results

**Figure 6:**
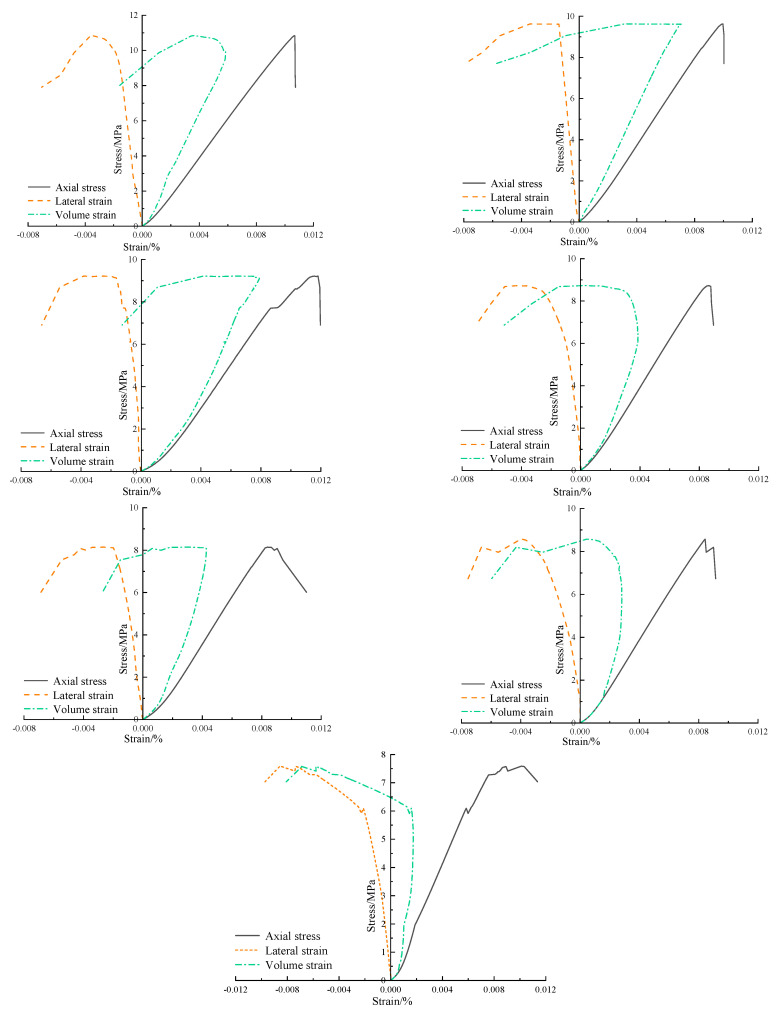
Stress–strain curves of coal samples with different soaking durations.

Mechanical tests were conducted on the coal samples with different soaking durations. Figure 6 shows the stress–strain curves for coal samples at representative soaking durations from each test group.

The stress–strain curves of coal samples with various soaking durations exhibit typical behavior in various stages, such as pore compaction, elastic deformation, micro-crack propagation, and post-peak failure. As the soaking duration increases, the post-peak failure response gradually transitions from brittle failure to ductile failure. This is consistent with the NMR test results, indicating that water infiltration increases the pore volume in coal samples and, in turn, prolongs the compaction stage. As the soaking duration increases, the coal sample failure mode shifts from brittle failure (unsoaked) to ductile failure (soaked for 56 days).

Processing the data on coal samples with different soaking durations yielded the mechanical parameters under different soaking durations (Table 2). Water considerably impacts the mechanical properties of the coal samples. Compared to the dry coal samples, the uniaxial compressive strength decreases continuously with the increasing soaking duration, with the average uniaxial compressive strength dropping from an initial 10.39 MPa to 8.06 MPa. The elastic modulus of the coal samples also decreases over longer soaking durations, with the average elastic modulus falling from 1.148 GPa to 0.924 GPa. Meanwhile, the Poisson’s ratio shows an increasing trend.

**Figure 7:**
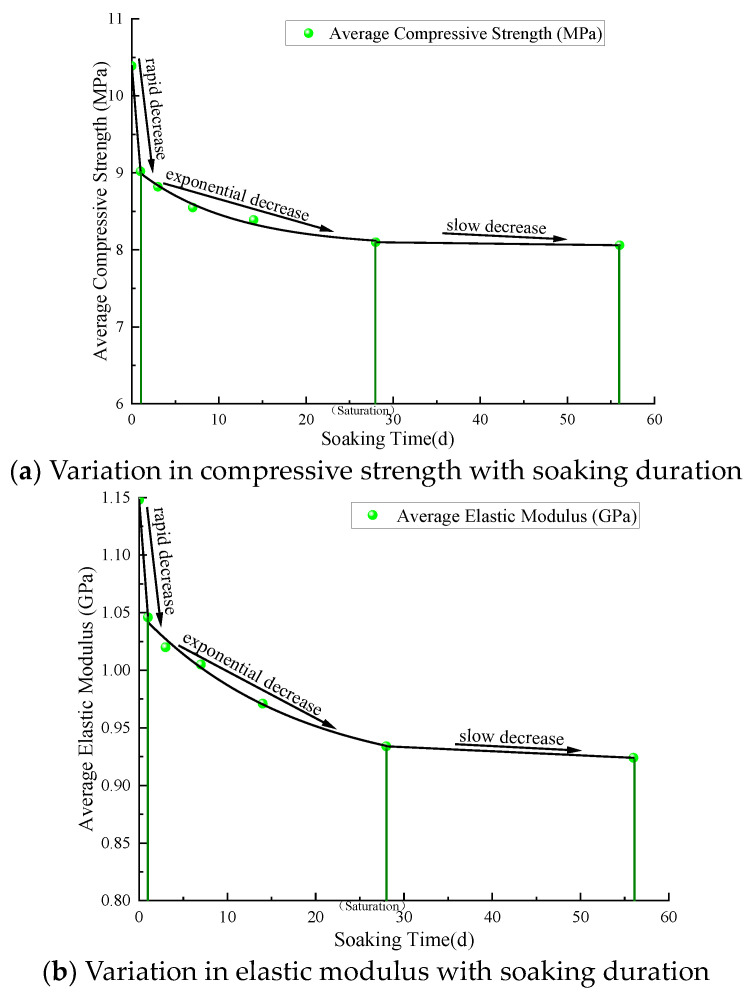
Variation in compressive strength (**a**) and elastic modulus (**b**) with soaking duration.

Segmented fitting was employed to further analyze the effect of soaking duration on coal strength, and Figure 7 shows the variation trends in compressive strength and elastic modulus of coal samples with soaking duration. In the first stage, the cleats and fractures along the coal seam collapse due to water infiltration and coal matrix swelling. The microscopic damage accumulates rapidly to deteriorate the macroscopic mechanical properties, manifested as rapid linear decreases in the compressive strength and elastic modulus of the coal samples. In the second stage, the compressive strength and elastic modulus exhibit exponential declines as water gradually diffuses deeper into the coal samples. These exponential decreases reflect the complexity and gradual nature of the water infiltration and diffusion process, with some slower physical and chemical processes beginning to take effect. The third stage features slower declines in compressive strength and elastic modulus. In this stage, water completely fills the pores inside the coal samples, and the weakening effect is primarily related to soaking duration. With longer soaking, the clay minerals in the coal expand further, and more soluble salts dissolve. Compared to the original coal samples, the compressive strength decreases by 13.2%, 10.2%, and 0.5% in the different soaking stages, while the elastic modulus decreases by 8.9%, 10.7%, and 1.1%, respectively.

#### 3.3.2. Analysis of Triaxial Test Results

To more comprehensively determine the weakening behavior of coal samples with different soaking durations, triaxial compression tests were conducted at confining pressures of 2 MPa and 4 MPa. The corresponding mechanical parameters are shown in Table 3.

The coal weakening behavior is characterized by the weakening coefficient (W, which is commonly applied to quantify the deterioration in the mechanical or physical properties of materials like coal under external influences (e.g., water immersion, heat treatment, and stress loading). The weakening coefficient is defined as the ratio of the post-immersion compressive strength of the coal sample (*R_w_*) to that of the dry sample (*R_d_*):

(3) $$W=\frac{R_{w}}{R_{d}}$$

The range of the weakening coefficient is 0 ≤ *W* ≤ 1, with a smaller value indicating a stronger weakening effect and a greater impact by water. In coal samples with different soaking durations, the weakening coefficient variation trend as the confining pressure increases is mainly influenced by the degree of microstructural damage caused by soaking, the strengthening effect of confining pressure on the mechanical properties, and the interaction between these two factors. Generally, the mechanical properties of coal tend to improve as the confining pressure increases. This is because the confining pressure limits the fracture expansion and pore deformation within the coal, reducing macroscopic damage due to fracture propagation. Therefore, the strengthening effect of the confining pressure partially offsets the weakening effect despite the micro-damage of soaking. As a result, the increased confining pressure suppresses the weakening effect of soaking, even though it is more pronounced in coal samples with longer soaking durations. This is manifested as an increased weakening coefficient, indicating that the coal properties gradually approach those of the unsoaked state.

However, the results from multiple tests differ from the common understanding. In the coal samples soaked for 1 day, the weakening coefficient initially increases and then decreases as confining pressure increases. Specifically, the weakening coefficients at confining pressures of 0, 2, and 4 MPa are 0.868, 0.893, and 0.881, respectively. This is because the strengthening effect of the confining pressure reaches its limit at 2 MPa, and further increases in confining pressure exacerbates internal damage, leading to smaller weakening coefficients.

In coal samples soaked for 3 to 56 days, the weakening coefficient decreases (indicating an enhanced weakening effect) as the confining pressure increases. This can be explained as follows. Compared to the initial stage with rapid water absorption, where water content increments serve as the main driver of coal microstructure destruction, the damage caused by minerals in the coal in the second and third stages does not fully manifest without external pressure. Although the evolution of those minerals causes some damage to the microstructure, its extent is limited by the skeleton formed by certain stable components, such as quartz and the coal matrix. As a result, the damage caused by soaking is not fully reflected in the macro-mechanical performance. However, the increasing confining pressure further activates the newly formed microcracks and pores in the coal, reducing the mechanical performance and enhancing the weakening effect. Hence, the smaller weakening coefficient. Therefore, the weakening coefficient decreases the most due to increased confining pressure in the third stage, with minerals dominating the coal damage, dropping from 0.779 at 0 MPa to 0.626 and 0.623 at 2 MPa and 4 MPa, respectively.

#### 3.3.3. Macroscopic Crack Propagation Patterns

**Figure 8:**
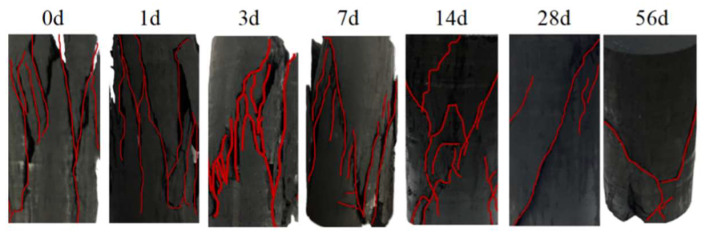
Failure modes of coal samples with different soaking durations.

The final failure modes of the coal samples from the uniaxial compression tests at different soaking durations are shown in Figure 8. The red lines represent the cracks on the coal sample surfaces.

The different macroscopic failure modes of the coal samples are often ascribed to internal microstructural changes [36]. Therefore, the crack propagation patterns at each stage are analyzed in detail in conjunction with the microstructural changes. With short-term soaking (0 to 1 day), the failure mode is primarily tensile failure. In the dry coal sample (soaked for 0 days), the numerous longitudinal tensile cracks on the surface, which are parallel to the loading axis, display typical tensile failure characteristics. The coal structure is relatively dense, with numerous cracks, mainly tensile, and no obvious shear cracks are observed. After 1 day of soaking, the coal sample still exhibits tensile failure as the dominant mode, with fewer cracks and shorter and shallower longitudinal cracks than the dry sample. Thus, short-term water infiltration affects the mechanical properties of the coal sample. However, tensile strength still dominates the failure mode, and no obvious shear failure occurs.

With 1 to 28 days of soaking, the failure mode of the coal samples gradually transitions from tensile failure to tensile–shear failure. After 3 days of soaking, shear cracks appear on the coal sample surfaces, interspersed with the longitudinal tensile cracks, constituting a composite failure mode. After 7 days of soaking, the shear cracks develop further, and the number of cracks decreases, though with persisting tensile cracks. After 14 days of soaking, the shear cracks saw more pronounced development with increased length and depth, and the tensile strength of the coal sample decreased largely. After 28 days, the number of cracks experiences a noticeable reduction. In addition, shear cracks dominate the failure process, while tensile cracks diminish, indicating a failure mode transition.

With long-term soaking (28 to 56 days), the failure mode of the coal samples is predominantly shear failure. After 56 days of soaking, only 2 to 3 distinct shear cracks appear on the coal sample surfaces, with a remarkably reduced number of cracks, and tensile cracks have almost disappeared. The coal samples maintain high integrity, with crack development largely confined to local areas, indicating a stabilized failure process with full dominance of shear failure.

The above analysis suggests that as soaking duration increases, the coal sample failure mode gradually shifts from tensile failure to tensile–shear failure and finally to shear failure. The primary reason for this transition lies in the continuous microstructural changes in the coal, particularly the mineral dissolution and expansion effects during soaking, which affect the internal bonding strength of the coal. In the initial soaking stage (0 to 1 day), the internal bonding of the coal sample is still strong, with a high friction coefficient and cohesion, resulting in the dominance of longitudinal tensile cracks. In the middle soaking stage (1 to 28 days), the soluble salt minerals (e.g., carbonate minerals) gradually dissolve into water, thus weakening the overall strength of the coal matrix, expanding microcracks, and promoting shear crack development. Meanwhile, the clay minerals (e.g., kaolinite, montmorillonite) undergo expansion due to water infiltration, further weakening the bonding between minerals, reducing the friction coefficient and cohesion, and resulting in a coexistence of tensile and shear cracks. Hence, the tensile–shear failure mode.

In the late soaking stage (28 to 56 days), the coal’s internal structure softens continuously, with the clay mineral expansion reaching saturation and the complete dissolution of soluble salt minerals, thus largely weakening the weak structural planes within the coal. At this stage, the tensile strength decreases sharply, and shear failure becomes dominant, with fewer cracks concentrated in local areas. Therefore, as soaking duration increases, the microstructural bonding strength of the coal continuously decreases, eventually leading to a failure mode shift from tensile failure to predominantly shear failure.

## 4. Discussions

This study systematically analyzed the changes in pore structure, mineral composition, and mechanical properties of coal samples under varying immersion durations. The findings revealed how water induces damage to the coal structure and mechanical properties. The results indicate that water immersion greatly alters the pore distribution and mineral composition of the coal, which profoundly impacts its mechanical behavior. These findings are discussed further, focusing on the effects of external factors and the chemical properties of water on coal damage mechanisms.

### 4.1. Mechanism of Coal Damage with Different Immersion Durations

**Figure 9:**
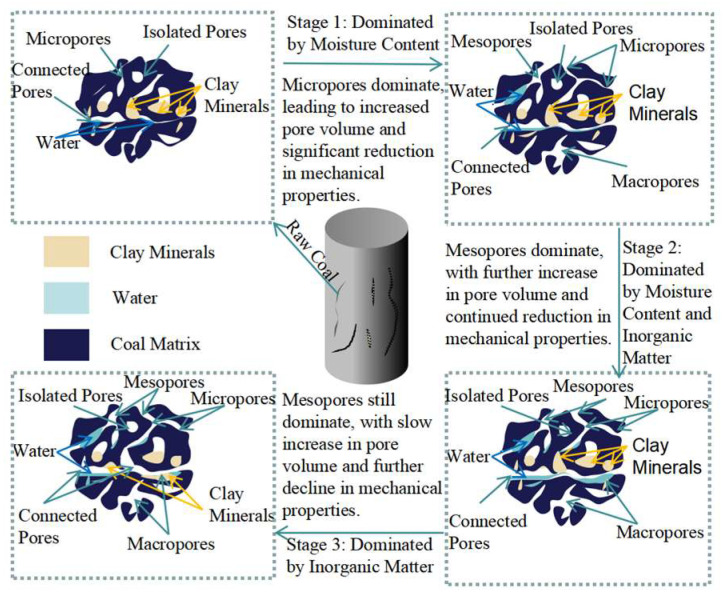
Evolution of coal pore structure at different soaking times.

The physical and chemical interactions between water and coal over long-term water immersion often weaken the mechanical strength of the coal. However, the primary water-coal interaction forms differ over extended soaking. Figure 9 illustrates the microstructural evolution of coal samples at each stage.

In the rapid water absorption stage, the primary effect is physical, manifested as rapidly increased water content and expanded pore-fracture structures. Water infiltration causes coal matrix swelling, changing its pore and fracture structures. This damage inevitably changes the coal’s macroscopic mechanical properties.

The uniform transition stage is markedly longer, and the coal tends to reach saturation. As the soaking duration increases, the water content slightly increases, and the pore volume growth rate is considerably lower than in the first stage.

In the stabilization stage, the coal is saturated, and the water-coal interactions are predominantly chemical. The swelling of clay minerals inside the coal and the dissolution of soluble salts further enlarge the pores. At this stage, the stable minerals and the coal matrix continue to serve as the structural skeleton, meaning that the weakening effect of water on the coal does not fully manifest. This is reflected in the minimal reduction in the weakening coefficient of coal under uniaxial conditions. However, the weakening effect becomes more pronounced under triaxial conditions, with remarkable weakening coefficient reductions compared to uniaxial conditions.

This alternating dominance of physical and chemical effects reveals the complexity of the weakening behavior of coal’s mechanical properties. In the early stages, the mechanical property weakening is mainly related to water content changes. After a long mid-term transition, the mechanical property weakening in the later stages is only related to soaking duration.

After water immersion, water molecules penetrate the original microcracks into the coal, rapidly infiltrating the interior through pores and fractures. During this process, the internal minerals undergo wetting, softening, and lubrication effects, thus reducing the cohesion and internal friction of the coal. Meanwhile, the internal minerals undergo chemical reactions, such as dissolution, diffusion, and migration. Pyrite in the coal undergoes oxidation in an oxygen-rich environment, generating sulfuric acid and ferric hydroxide, and the chemical reaction is as follows:

(4) $$4{\mathrm{FeS}}_{2}+15O_{2}+14H_{2}O\to 4\mathrm{Fe}{\left(\mathrm{OH}\right)}_{3}+8H_{2}{\mathrm{SO}}_{4}$$

The sulfuric acid generated from pyrite oxidation leads to coal acidification, which promotes the dissolution of other minerals and, in turn, increases the porosity. Calcite dissolves in water and reacts with carbon dioxide, and the chemical reaction is as follows:

(5) $${\mathrm{CaCO}}_{3}+H_{2}O+{\mathrm{CO}}_{2}\to {\mathrm{Ca}}^{2+}+2{\mathrm{HCO}}_{3}^{-}$$

Kaolinite, a lamellar clay mineral, has hydrophilic properties on its gibbsite surface, while its siloxane surface is hydrophobic. Kaolinite particles aggregate tightly through hydrogen bonding, but its hydrophilic surface enables rapid water absorption at high temperatures, leading to expansion and volume increase. Montmorillonite has a layered structure, with each layer adsorbing a large number of water molecules, causing pronounced swelling upon water infiltration.

The chemical reactions and physical swelling effects of the inorganic minerals in coal, induced by irreversible deformation, cause stress concentration in the internal microcracks, thus forming new microcracks or activating existing ones. The additional water molecules provide more passages for water diffusion and movement. The physical swelling and chemical dissolution of the minerals, together, weaken the mechanical properties of the coal. Macroscopically, this results in decreased compressive strength and elastic modulus.

### 4.2. Effects of External Factors on Water-Induced Coal Damage

In addition to immersion duration, factors like geostress, temperature, coal type, and water’s chemical properties (e.g., salinity and pH) largely affect the water-induced damage to coal. According to Lyu et al. [37], geostress compacts the pore structure in coal and inhibits pore expansion. However, a higher geostress may induce shear failure, altering the pore structure and permeability of the coal. Furthermore, Qi et al. [38] conducted freeze-thaw tests with liquid nitrogen and found that temperature variations notably impact the pore structure evolution in coal. At higher temperatures, the hydrochemical reaction rate increases, accelerating mineral dissolution and pore expansion. These findings suggest that future research should consider the combined effects of temperature and geostress on the water-damage mechanisms in coal so that the coal behavior in actual coal seam environments can be more accurately simulated.

Coal samples with varying coalification degrees exhibit clearly different changes in pore structure and permeability during water immersion. Wang et al. [39] found that the mineral composition and clay content of coal considerably affected its pore expansion and structural weakening during hydration. In particular, coal samples with high clay contents tend to swell in water, leading to reduced overall strength. These findings provide a basis for further investigation into the water damage mechanisms in different coal types, supporting more precise coal hydration response predictions and applications in practical engineering.

Additionally, the chemical properties of water play a crucial role in water-induced damage. Luo et al. [40] demonstrated that the salinity and pH of water have pronounced chemical effects on coal hydration. High salinity and acidity intensify mineral dissolution and ion exchange reactions, which greatly impact pore connectivity and coal stability. Based on the results of this study and Luo Jinzhi’s findings, regulating water salinity and pH may offer a means to optimize coal seam water injection and hydraulic fracturing strategies, thereby enhancing permeability and improving coal seam stability.

### 4.3. Limitations and Future Prospects

This study revealed the effects of immersion duration on the pore structure and mechanical properties of coal samples under controlled experimental conditions. However, the impact of water-induced damage is more complex in actual coal seam environments due to the interplay of multiple factors, such as temperature and geostress. Future research should examine coal-water interactions across various coalification stages and under different stress and temperature conditions to more accurately simulate the water damage mechanisms in real-world coal seams.

Additionally, this study did not address the systematic regulation of water’s chemical properties. Future experiments could explore the effects of adding high-salinity water or adjusting the pH levels to assess coal damage under diverse chemical conditions. Applying numerical simulation tools to model the changes in coal permeability and mechanical properties under different stress, temperature, and chemical conditions could help reveal the multi-scale effects of water damage on coal. Such research could provide theoretical support and optimization strategies for coal seam water injection and hydraulic fracturing practices.

## 5. Conclusions

(1) The damage and weakening processes of water immersion on the coal samples are investigated. The weakening process transitions gradually from physical infiltration to chemical dissolution, encompassing the rapid water absorption, uniform transition, and stabilization stages. In the initial stage, water quickly infiltrates pores and micro-cracks, triggering matrix swelling and micro-crack expansion through physical infiltration. During the transition stage, the coal sample approaches saturation, the infiltration rate decreases, the pore expansion stabilizes, and the dominant damage mechanism shifts gradually to chemical dissolution. In the stabilization stage, chemical dissolution and mineral expansion become prominent, further expanding the pores. The alternating physical and chemical effects determine the evolution of the coal’s microstructure and macroscopic mechanical properties.
(2) The compressive strength and elastic modulus of the coal decrease substantially with the increasing soaking duration. During the rapid water absorption stage, the mechanical properties of the coal deteriorate rapidly, with the compressive strength decreasing linearly. In the uniform transition stage (soaked for 1 to 28 days), the mechanical properties deteriorate exponentially. In the stabilization stage (after 28 days), the compressive strength and elastic modulus decline slowly. The proportion of pore volumes remains stable, with no pronounced pore transformation observed, and the damage is mainly ascribed to mineral dissolution and swelling.
(3) Soaking duration largely impacts the macroscopic failure modes of the coal samples. Unsoaked and briefly soaked coal samples primarily exhibited tensile failure, with more cracks and distinct longitudinal fractures. As the soaking duration increases, tensile–shear failure appears, and the number of cracks decreases. Soaked for a long time (28 and 56 days), shear cracks become dominant, with fewer cracks and better structural integrity.
(4) XRD analysis shows that clay minerals, such as montmorillonite and kaolinite, cause an increase in pore volume during water absorption and swelling, weakening the coal structure. Meanwhile, the dissolution of pyrite and calcite further exacerbates pore expansion and pore network formation, leading to continuous degradation of the coal’s mechanical properties. The mineral swelling and dissolution effects do not fully manifest under uniaxial conditions in the stabilization stage. The weakening coefficient decreases slowly under uniaxial conditions but markedly with the increasing confining pressure, thus enhancing the weakening effect.

## Figures and Tables

**Table 1:** Quantitative data on the mineral composition of coal samples soaked for various durations.

Soaking Time (days)	Mineral Composition (%)				
	Kaolinite	Quartz	Pyrite	Montmorillonite	Calcite
0	48.7	21.9	17.4	5.9	6.1
1	58.2	19.8	8.9	7.2	5.9
3	60.2	19.4	7.5	6.8	6.1
7	41.3	40.2	7.2	6.0	5.3
14	41.5	43.5	5.9	5.1	4.0
28	44.5	45.4	3.1	3.4	3.6
42	53.2	42.8	1.2	1.0	1.8
56	77.2	22.6	0.2	-	-

**Table 2:** Mechanical properties of coal samples at different soaking times.

Soaking Duration (Days)	Sample ID	Compressive Strength (MPa)	Average Compressive Strength (MPa)	Elastic Modulus (GPa)	Average Elastic Modulus (GPa)	Poisson’s Ratio	Average Poisson’s Ratio	Weakening Coefficient (W)
0	D-0-1	10.84	10.39	1.118	1.148	0.143	0.126	1.000
	D-0-2	10.14		1.125		0.068		
	D-0-3	10.19		1.201		0.167		
1	D-1-1	9.63	9.02	1.069	1.046	0.089	0.159	0.868
	D-1-2	8.68		1.022		0.214		
	D-1-3	8.75		1.048		0.175		
3	D-3-1	9.22	8.82	1.033	1.020	0.155	0.191	0.849
	D-3-2	8.63		1.012		0.198		
	D-3-3	8.61		1.016		0.221		
7	D-7-1	8.73	8.55	1.041	1.005	0.24	0.256	0.823
	D-7-2	8.44		1.004		0.243		
	D-7-3	8.48		0.969		0.285		
14	D-14-1	8.58	8.39	1.001	0.971	0.303	0.276	0.808
	D-14-2	8.13		0.906		0.241		
	D-14-3	8.45		1.007		0.285		
28	D-28-1	8.15	8.10	0.984	0.934	0.221	0.281	0.780
	D-28-2	8.18		0.917		0.295		
	D-28-3	7.98		0.902		0.328		
56	D-56-1	7.59	8.06	0.920	0.924	0.201	0.305	0.779
	D-56-2	8.47		0.937		0.393		
	D-56-3	8.11		0.916		0.321		

**Table 3:** Triaxial test results of coal samples with different soaking durations.

Soaking Duration (Days)	Sample ID	Confining Pressure (MPa)	Compressive Strength (MPa)	Weakening Coefficient (W)
0	S-0-1	2.00	16.24	1.000
	S-0-2	4.00	18.83	1.000
1	S-1-1	2.00	14.50	0.893
	S-1-2	4.00	16.58	0.881
3	S-3-1	2.00	13.32	0.820
	S-3-2	4.00	14.85	0.789
7	S-7-1	2.00	12.15	0.748
	S-7-2	4.00	13.94	0.740
14	S-14-1	2.00	11.67	0.719
	S-14-2	4.00	12.67	0.673
28	S-28-1	2.00	11.27	0.694
	S-28-2	4.00	12.39	0.658
56	S-56-1	2.00	10.16	0.626
	S-56-2	4.00	11.74	0.623

## Data Availability

The original contributions presented in the study are included in the article, further inquiries can be directed to the corresponding author.

## References

[1] Kang H.-P. (2020). Spatial scale analysis on coal mining and strata control technologies. J. Min. Strat. Control Eng..

[2] Chomać-Pierzecka E., Gąsiński H., Rogozińska-Mitrut J., Soboń D., Zupok S. (2023). Review of Selected Aspects of Wind Energy Market Development in Poland and Lithuania in the Face of Current Challenges. Energies.

[3] Liu Z.-G., Qiao G.-D., Liu J., Gao K. (2024). Research progress and prospect of coal seam blasting antireflection technology in China. J. China Coal Soc..

[4] Cheng J.-J., Liu Y. (2024). Coal mine rock burst and coal and gas outburst image perception alarm method based on depth characteristics. Coal Sci. Technol..

[5] Yue J.-W., Han Q.-J., Liang Y.-H., Shi B.-M. (2024). Dynamic evolution characteristics and microscopic mechanisms of contact between gas-bearing coal and water. Coal Sci. Technol..

[6] Shan W.-X., Chen X.-X. (2024). Study on the gas outburst control effect of goaf on the neighboring working face: Case study of Pingmei No.4 Mine. Energy Sci. Eng..

[7] Li T., Zhang H.-W., Han J. (2011). Controlling effect of tectonic stress field on coal and gas outburst. J. Xi’an Univ. Sci. Technol..

[8] Li T., Zhang J.-W., Jin Z.-P. (2017). Numerical research into solid-gas-thermal coupling of coal and rock containing gas. J. Heilongjiang Univ. Sci. Technol..

[9] Zhang X.-G., Jiang W.-Z., Du F. (2021). Development status and prospect of permeability enhancement technology in high gas low permeability coal seam. Saf. Coal Mines.

[10] Lu Y.-Y., Huang S., Ge Z.-L. (2022). Research progress and strategic thinking of coal mine water jet technology to enhance coal permeability in China. J. China Coal Soc..

[11] Yuan L., Lin B.-Q., Yang W. (2015). Research progress and development direction of gas control with mine hydraulic technology in China coal mine. Coal Sci. Technol..

[12] Zhuang X.-W., Tang C., Wu H.-T. (2023). Influence of moisture on seepage characteristics of coal during total stress-strain process. Saf. Coal Mines.

[13] Lu W.-Y., Liu Q., Qu L.-N. (2022). Study on coal crack propagation and failure mode with different moisture content under uniaxial compression. J. Mine Autom..

[14] Han P.-H., Zhao Y.-X., Gao S. (2024). Progressive damage characteristics and damage constitutive model of coal samples under long-term immersion. Chin. J. Rock Mech. Eng..

[15] Zhang C., Jia S., Wang F.-T. (2023). An Experimental Research on the Pore and Fracture Evolution Characteristics and Its Driving Mechanism for Coal Samples Under Water-rock Interaction. J. Basic Sci. Eng..

[16] Zhang H.-M., Xia H.-J., Zhang J.-F. (2024). Damage evolution mechanism of coal rock under long-term soaking. Chin. J. Geotech. Eng..

[17] Qin L., Ke S.-H., Li S.-G. (2023). Characteristics of unfrozen water content and its influence on pores of liquid nitrogen cyclic frozen coal during thawing process. J. China Coal Soc..

[18] Du Q., Liu X., Wang E., Zuo J., Wang W., Zhu Y. (2020). Effects of CO2-water interaction with coal on mineral content and pore characteristics. J. Rock Mech. Geotech. Eng..

[19] Zhai C., Sun Y., Fan Y.-R. (2022). Application and prospect of low-field nuclear magnetic resonance technology in accurate characterization of coal pore structure. J. China Coal Soc..

[20] Guo G.-S., Feng L.-R., Li H. (2020). Quantitative characterization of different coal rank reservoirs permeability based on NMR and X-CT technology. Geol. Surv. China.

[21] Feng X.-Z. (2018). Nuclear magnetic resonance investigation on the mechanism of water damage to mudstone. Sci. Technol. Eng..

[22] Reyila A., Ma F.-Y., Zhang X. (2017). Application of low-field nuclear magnetic resonance technology in coal petrographic pore structure. Nucl. Tech..

[23] Chen J., Sun Y., Ling Y., Chu X., Cheng Y., Min F. (2024). Effects of Mg(II) doping amount on the hydration characteristics of kaolinite surface: Molecular dynamics simulations and experiments. Surf. Interfaces.

[24] Zhang S., Liu Q.-F., Cheng H.-F., Liu C. (2018). Mechanism responsible for intercalation of dimethyl sulfoxide in kaolinite: Molecular dynamics simulations. Appl. Clay Sci..

[25] Ko L.T., Loucks R.G., Rowe H., Adriaens R., Sivil J.E., Mertens G. (2024). Mudstone diagenesis with depth and thermal maturity in the Cenomanian-Turonian Eagle Ford group. PART II: Diagenetic processes and paragenetic sequence. Mar. Pet. Geol..

[26] Gregerová M., Štulířová J., Frýbort A., Grošek J. (2024). Impact of light mica on the intensity of the alkali-silica reactions in cement concrete pavements containing cataclased granite aggregates. Case Stud. Constr. Mater..

[27] Giovanni G., Agim S., Micol B., Giuseppe C., Alessandro C. (2021). Environmental Impact Variability of Copper Tailing Dumps in Fushe Arrez (Northern Albania): The Role of Pyrite Separation during Flotation. Sustainability.

[28] Falzone S., Keating K. (2016). A laboratory study to determine the effect of pore size, surface relaxivity, and saturation on NMR T2 relaxation measurements. Near Surf. Geophys..

[29] Luo Z.-X., Paulsen J., Song Y.-Q. (2015). Robust determination of surface relaxivity from nuclear magnetic resonance DT 2 measurements. J. Magn. Reson..

[30] Xu J., Xu H., Zhai C., Cong Y., Sang S., Ranjith P., Li Q., Ding X., Sun Y., Lai Y. (2023). Surface relaxivity estimation of coals using the cutting grain packing method for coalbed methane reservoirs. Powder Technol..

[31] Yang M., Zhang T., Zhang X.-B., Xu J., Han L.-X., Ma J. (2023). Study on the effect of NMR-based surfactants on pore wetting of high-order coal. Coal Sci. Technol..

[32] Chen S.-G., Zhang H.-M., Cheng S.F., Wang L., Yuan C., Li Z.-L. (2024). Identification of interfacial influence zone (IIZ) in clay rock-cement mortar binary based on the macro-pore distribution in space: An NMR and CT investigation. Mater. Lett..

[33] Tang L.Y., Li Y.-H., Yu Y.-T., Jin L., Gao Z.-G., Wu D., Sun Q., Jia H.-L., Luo T. (2024). Strength Deterioration Mechanism of Interface between Soil–Rock Mixture and Concrete with Different Degrees of Roughness under Freeze–Thaw Cycles. J. Cold Reg. Eng..

[34] Mondal I., Singh H.-K. (2024). Petrophysical insights into pore structure in complex carbonate reservoirs using NMR data. Pet. Res..

[35] Xi Y., Xing J.-H., Wang H.-J., Wang W., Li J., Fan L.-F. (2024). Evaluation of pore characteristics evolution and damage mechanism of granite under thermal-cooling cycle based on nuclear magnetic resonance technology. Geoenergy Sci. Eng..

[36] Dou L., Cao J., Cao A., Chai Y., Bai J., Kan J. (2021). Research on types of coal mine tremor and propagation law of shockwaves. Coal Sci. Technol..

[37] Lyu M.-J., Liu L.-R., Zhang X.-T., Chen B. (2024). Dynamic evolution law of coal seepage under hydraulic coupling. J. Min. Strat. Control Eng..

[38] Qi X.-H., Zhang Y., Hou S.-R., Liu X.-D., Liu Z.-H., Xie W.-K. (2024). Study on Mechanical and Seepage Characteristics of Liquid Nitrogen Freeze-Thaw Coal with Different Water Content. Chin. Q. Mech..

[39] Wang F.-T., Zhang C., Tang T.-K., Jia S., Cheng J., Dou F. (2024). Pore and strength damage evolution mechanism of coal induced by the circulating water immersion effect. J. Min. Sci. Technol..

[40] Luo J.-Z., Tang H., Sui Z.-L. (2021). Hydrochemistry of coal samples in water immersion process. Sci. Technol. Eng..

